# Population-Scale Foraging Segregation in an Apex Predator of the North Atlantic

**DOI:** 10.1371/journal.pone.0151340

**Published:** 2016-03-22

**Authors:** Vitor H. Paiva, Ana I. Fagundes, Vera Romão, Cátia Gouveia, Jaime A. Ramos

**Affiliations:** 1 MARE – Marine and Environmental Sciences Centre, Department of Life Sciences, University of Coimbra, 3004–517 Coimbra, Portugal; 2 SPEA – Sociedade Portuguesa para o Estudo das Aves, Travessa das Torres 2A 1°, 9060–314 Funchal, Madeira, Portugal; Phillip Island Nature Parks, AUSTRALIA

## Abstract

In this work we investigated the between-colony spatial, behavioural and trophic segregation of two sub-populations of the elusive Macaronesian shearwaters *Puffinus baroli* breeding only ~340 km apart in Cima Islet (Porto Santo Island) and Selvagem Grande Island. Global location sensing (gls) loggers were used in combination with the trophic ecology of tracked individuals, inferred from the isotopic signatures of wing feathers. Results suggest that these two Macaronesian shearwater sub-populations do segregate during the non-breeding period in some ‘sub-population-specific’ regions, by responding to different oceanographic characteristics (habitat modelling). Within these disparate areas, both sub-populations behave differently (at-sea activity) and prey on disparate trophic niches (stable isotope analysis). One hypothesis would be that each sub-population have evolved and adapted to feed on particular and ‘sub-population-specific’ resources, and the segregation observed at the three different levels (spatial, behavioural and trophic) might be in fact a result of such adaptation, from the emergence of ‘cultural foraging patterns’. Finally, when comparing to the results of former studies reporting on the spatial, behavioural and trophic choices of Macaronesian shearwater populations breeding on Azores and Canary Islands, we realized the high ecological plasticity of this species inhabiting and foraging over the North-East Atlantic Ocean.

## Introduction

The use of miniaturized tracking devices (such as global location sensing—gls—devices) in combination with Stable Isotope Analysis (SIA), has become a powerful tool to study in an holistic manner, the spatial and trophic segregation of marine apex predators. SIA is based on the assumption that the isotopic signature of predators is directly influenced by what they consume [1]. Thus, the stable carbon signature of consumers is similar to that of their diets, therefore differing among foraging locations and making it a useful tool for the study of spatial segregation in foraging regions. The nitrogen signature reflects the predators’ trophic position, with a stepwise increase at each trophic level and allows the investigation of trophic segregation among predators. Furthermore, because animal tissues are synthetized in a predictable manner and have different turnover rates, we can investigate the consumers’ dietary choices from the previous weeks (whole blood) to months (new growing feathers after moult; [2]).

Marine top predators generally forage in areas where physical and oceanographic processes (e.g. seamounts or mainstream oceanic currents) enhance productivity and congregate potential prey [3]. Ocean circulation in the North Atlantic is influenced by a major gyre; a circular current that is confined on one side by the North American and Canadian coastlines, and on the other by the Portuguese and African coasts [4]. Productivity on the east side of this gyre, is majorly influenced by the Azorean Current (which descends from the Labrador Current, joins the Gulf Stream and crosses Azorean islands) and the Portuguese Current, descending along the mainland Portugal towards the African coast, where it forms the Canary Current [5]. This coastal movement, in conjunction with the highly sloped shelf break and wind transport of surface water, promotes the strong upwelling of cold, nutrient-rich waters off both the Portuguese [6] and African [7] coasts. The upwelling along these Current systems enriches those areas and the amount of phytoplankton and zooplankton increases, followed by an increase of small pelagic fishes, and consequently the presence of relevant populations of seabird (e.g. [7]), predatory fish (e.g. [8]), cetacean (e.g. [9]), and sea turtle (e.g. [9]) species, and provide resources to an intensive industrial fishery at the area all-year round [10]. Despite all the potential conflicting situations between “predators” feeding on similar food sources, especially between Humans (fisheries) and marine animals (e.g. seabirds), there are few monitoring data available for the region (but see [7]) compared to other upwelling systems of the world (e.g. the California Current; [11]).

Most research on the relationships between seabirds and the marine environment has focused on large seabird species, which are easier to follow using tracking devices. In the north Atlantic, in particular, larger seabirds such as Cory’s shearwater *Calonectris borealis* have been widely researched (e.g.[7]) but smaller species such as the Macaronesian Shearwater *Puffinus baroli*, have been largely neglected (but see [12,13]). This species is observed around its colonies throughout the year, which suggest a non-migratory behaviour, and the necessary adaptation to locate profitable food patches all-year round at the same relatively confined region. Overall, very limited information exists about the breeding biology, life cycle phenology and at-sea yearly distribution of Macaronesian shearwaters from the North Atlantic (but see [12,14]). Birds of the Azores population lay their egg in late January, eggs hatch in mid-March and chicks fledge in late May. Adults are usually absent from the breeding colonies between June and September [15].

We investigated the between-colony spatial, behavioural and trophic segregation of two sub-populations of Macaronesian shearwaters breeding only ~340 km apart in Cima Islet (CI; Porto Santo Island) and Selvagem Grande Island (SG). CI should be home of a population of 150–300 breeding pairs (~3–5% of the total population of Macaronesian shearwater) (Natural Park of Madeira unpublished data), and SG should hold 1500–4000 breeding pairs (roughly 40–60% of the total population) [16], both colonies thus hold between 45–75% of the total species population. The remain species populations occur in the Azores, which should hold between 840 and 1530 pairs [17], whereas the Canary Islands should be home for ~400 breeding pairs [18]. However, more recent data concerning population size from all enunciated breeding places is still lacking. Recent work by Ramos et al. [13], shed some light over the feeding (diet composition) and trophic (SIA) ecology of both populations in two years of contrasting environmental conditions. In general, both populations feed mostly on cephalopods, but increased the consumption of fish and crustaceans when environmental conditions were better. The ameliorating conditions, also narrowed the isotopic niche of both populations, with both carbon and nitrogen signatures being generally higher for CI when compared to SG individuals.

In this work, global location sensing (gls) loggers were used in combination with the trophic ecology of tracked individuals, inferred from the isotopic signatures of wing feathers. Previous studies with Cory’s shearwater breeding at the same study places (e.g. [7]), suggest that the very productive Canary Current (CC) ecosystem is intensively exploited by local seabird populations. Therefore, we hypothesized that such area should also be relevant for Macaronesian shearwaters throughout the year. More specifically, we expect that: (A) while breeding, the spatial, behavioural and trophic segregation between colonies should be low, because birds become ‘central-place foragers’ and thus need to find food resources within the colony surroundings or nearby their breeding locations, like within the CC ecosystem. The at-sea behavioural patterns and, until some extent, dietary choices should be mostly dictated by their colony duties and therefore, we expect a general resemblance in the foraging ecology of CI and SG sub-populations; (B) When relieved from their breeding duties, a higher spatial segregation among CI and SG birds should exist, which will necessarily lead to differences in their trophic and behavioural ecology. This meaning, birds from CI and SG sub-populations might feed on isotopically different local prey and strategies used to capture it may differ. These expectations for the trophic ecology are in line with results provided by Ramos et al. [13], where both populations showed a larger isotopic niche during the non-breeding phase, when compared to the breeding period. Meaning that during non-breeding, the populations likely exploited an enlarged array of prey species within different ‘population-specific’ isoscapes.

## Methods

### Ethics statement

The deployment of MK18L loggers (see details below) did not take more than 10 minutes and on no occasion did it interfere with reproduction or have visible deleterious effects on study animals. All work was approved and certified by annual permits by the relevant authorities, the Service of the Natural Park of Madeira.

### Fieldwork and tracking

Between 2010–2014, global location sensing (gls) loggers (MK18L –BAS company) were deployed and recovered from Macaronesian shearwaters breeding in Selvagem Grande Island (SG, N = 9 yearly trips from 8 individuals) and in Porto Santo Island, Cima Islet (CI, N = 14 yearly trips from 10 individuals). Ten devices were deployed each year (2010–2012) at each breeding colony, and we recovered 4, 2 and 2 from SG and 3, 4 and 3 from CI, respectively in 2011, 2012 and 2013. In 2012, one individual from SG was tracked during 2 consecutive years and in 2013 two birds from CI were also tracked during two consecutive years and one during 3 years. Only successful breeders were tracked, to control for the effect of the absence of breeding duties on the spatial and behavioural patterns being accessed [19]. Loggers were attached with a cable tie to the numeric metallic ring and represented ~1% of the bird’s weight. Upon recovery, feather (innermost primary and eight secondary) samples were collected from all tracked individuals for stable isotope analysis (trophic ecology). Also, a blood sample (~50 μl) was taken from the bird’s tarsal vein for molecular sexing following the methods by [20] after DNA had been extracted using an adaptation of the Chelex method [21]. The process didn´t took more than 10 minutes per individual. All data are available at the BirdLife International seabird tracking database (http://www.seabirdtracking.org) at the following addresses http://seabirdtracking.org/mapper/?dataset_id=1028 and http://seabirdtracking.org/mapper/?dataset_id=1029. Data are also available on figshare (https://dx.doi.org/10.6084/m9.figshare.3112639.v1).

The devices recorded light (for geolocation) and salt-water immersion (for analysis of activity) data. Geolocation is the calculation of position (twice per day) from ambient light level readings with reference to time. Latitude and longitude were estimated from day (night) length and the time of local noon (mid-night), respectively, in relation to Greenwich time [22]. Data were analyzed using the *BASTrack* software suite (British Antarctic Survey, Cambridge, UK), using a light threshold of 10 and with elevation angles of −4.0 and -4.5 (derived from calibration devices left at Cima Islet and Selvagem Grande, respectively). The quality of the light curves checked with *TransEdit* was high, so the geolocation error was assumed to be similar to that estimated by [22]. Locations derived from curves with apparent interruptions around sunset and sunrise were removed. Erroneous locations were also excluded for a week around the equinoxes, when latitudes are unreliable. Overall, nearly 87% of the original locations were retained as valid geolocation estimations. Validated data were smoothed twice [22].

### At-sea activity

The activity patterns of Macaronesian shearwater were derived from both immersion and light level data recorded for each bird. The loggers tested for salt- water immersion every 3 s using 2 electrodes and stored the number of positive tests from 0 (continuously dry) to 200 (continuously wet) at the end of each 10 min period. The loggers also measured light level every minute and stored the maximum (truncated at a value of 64) at the end of each 10 min period. Each 10 min block was categorized as daylight and darkness, based on the timing of nautical twilight (derived by interpreting light curves in *TransEdit*; *BASTrack* software) following [23]. The immersion data were then categorized into day and night (based on the light data) representing the proportion of time spent on the sea surface (as distinct from flying or on land) during daylight and darkness. Time budget calculations excluded periods spent in burrows (prolonged—more than 40 min.–periods of darkness and dry records or dark periods during the day). Periods that the birds spent on the water surface were identified as any continuous sequence of 10 min blocks with at least 3 s sitting on the water, while a continuous sequence of dry (0) values was considered as a flight bout. Light and activity (immersion) data were used simultaneously to distinguish time spent at sea from time in the colony (burrows) and hence colony attendance patterns. These patterns and the duration of foraging trips, identified based on the logger data, were also confirmed on the ground by monitoring the burrow visits of the tracked individuals. These data were analyzed using customised functions and functions within the *adehabitatHR* package [24] in the R environment [25] to extract accurate information on at-sea activity patterns and the timing of breeding events.

Two different periods were considered as major phases of the yearly life cycle of the species and were used as units of analysis throughout the work: (1) the breeding (roughly between December and May) and (2) the non-breeding (between June and November) periods. The previous phases were identified checking both location and the light (*.lig) and activity (*.act) datasets within R (i.e. mainly using several functions inside the *adehabitatHR* package; [24]).

### Oceanographic predictors

To characterize the oceanographic conditions in areas used by the tracked individuals we extracted: (1) Bathymetry (BAT, blended ETOPO1 product, 0.01° spatial resolution, m), (2) Sea Surface Temperature (SST, Aqua MODIS NPP, 0.04°, °C), (3) sea surface Chlorophyll *a* concentration (CHL, Aqua MODIS NPP, 0.04°, mgm^-3^), gradients in these 3 variables–(4) BATG, (5) SSTG and (6) CHLG, respectively—and (7) wind speed (WSPD, QuickSCAT, 0.12°, ms^-1^). All variables were downloaded from the BloomWatch website (http://coastwatch.pfeg.noaa.gov/coastwatch/CWBrowserWW180.jsp) except for WSPD which was extracted from the SeaWinds database (http://winds.jpl.nasa.gov). Monthly averages were used for the dynamic variables (variables 2, 3 and 5–7). Gradients were determined by estimating rates of change by moving a window function (3 x 3 grid cells; function = [(max. value − min. value) × 100] / (max. value)). Fronts, as zones of strong CHL variations, will appear more clearly when using CHLG than using CHL values alone. Gradient in depth (BATG) was used as a proxy of slope. All environmental predictors were gathered to the coarsest grid cell (0.25°). This is more accurate than the geolocation data, which have an error of c. 180 km. However, previous studies suggest that neither a change in grain size nor locational errors significantly affected predictions from species distribution models (e.g. [26]).

### Habitat use

Tracks were obtained from the *Locator* program (inside BASTrack software). Predicted geolocations of each bird were examined under the *adehabitatHR* R package [24] generating Kernel Utilization Distribution (Kernel UD) estimates with a smoothing parameter (*h*) of 1° and a grid size of 0.25° (matching the grid of environmental predictors). The *h*-value approximates the mean accuracy of these devices [22] and thus has been used by other authors (e.g. [27]). Following previous authors (e.g. [28]), we considered the 50% and 95% kernel density contours to represent the core area of activity and the area of active use, respectively. The overlap between kernel UDs of (1) different individuals within a population and (2) between populations were computed to study the spatial segregation within and among groups with the *kerneloverlap* function of the *adehabitatHR* library.

### Habitat suitability models

#### Data processing and exploratory analysis

To minimize the influence of any particular individual on each model, we randomly selected an equal number of locations for each bird during a specific phase (i.e. breeding and non breeding period), based on a bootstrapping procedure [29]. All 8 predictor variables for each breeding stage were inspected under MaxEnt Model Surveyor (MMS; http://phycoweb.net/software/MMS/index.html), which automatically computed the Akaike and Bayesian information criteria (AIC, BIC; [30]) and the test AUC under the various predictor sets and suggested "suitable" predictor sets for our dataset [31], thus avoiding including highly correlated variables on our models.

#### Model evaluation and calibration

Model construction, training and testing was performed with Maximum Entropy (MaxEnt) modelling based on presence-only data ([32]; version 3.3.3 (http://www.cs.princeton.edu/~schapire/maxent/). The MaxEnt method does not require absence data for the species being modelled; instead it uses background environmental data from the entire study area. This method has been shown to perform well in comparison with alternative methods [33] and when modelling habitat use from tracking data (e.g. [34–36]). The tracking data was divided into train and test data by setting aside approximately 30% of the tracking dataset for spatial evaluation of the models [37]. We ran MaxEnt on the presence-only positions 50 times. The mean of the 50 MaxEnt predictions was calculated to obtain an average prediction and coefficient of variation of predictions [29]. The MaxEnt program was run separately for the breeding and non-breeding periods. The settings were logistic output format, resulting in values between 0 and 1 for each grid cell, where higher values indicate more similar climatic conditions, duplicates removed, and 50 replicate runs of random (bootstrap) subsamples with 30 as random test percentage. The results were summarized as the average of the 50 models.

From the MaxEnt main results, the Jackknife chart was used to evaluate the contribution of each environmental layer to the final result, thus providing the explanatory power of each variable when used in isolation. The ROC curve was used to assess the model’s accuracy, as measured by the Area Under the ROC Curve (AUC). The AUC estimates the likelihood that a randomly selected presence point is located in a raster cell with a higher probability value for species occurrence than a randomly generated point [32]. Generated models are generally interpreted as excellent for test AUC > 0.90, good for 0.80 < AUC < 0.90, acceptable for 0.70 < AUC < 0.80, bad for 0.60 < AUC < 0.70 and invalid for 0.50 < AUC < 0.60. All model evaluation statistics and optimal thresholds were calculated using the package *PresenceAbsence* in R [25].

### Trophic ecology

We collected portions of the innermost primary (P1) and the eighth secondary (S8) from each of the tracked birds (upon logger removal) to calculate the content in carbon (δ^13^C, ^13^C/^12^C) and nitrogen (δ^15^N, ^15^N/^14^N) stable isotopes. Sample sizes corresponded to the amount of devices retrieved each year, with 4, 2 and 2 feather samples from SG and 3, 4 and 3 from CI, respectively in 2011, 2012 and 2013. Feather samples were stored in polythene bags until stable isotope analysis (SIA). Isotopic ratios were determined by continuous-flow isotope-ratio mass spectrometry (CF- IRMS). In Macaronesian shearwaters, primary feathers moult during April—May and therefore should represent the trophic ecology of the individual during the end of the previous breeding period. Secondary feathers moult in October-November, thus representing the end of the previous non-breeding period [14].

### Statistical analysis

Generalized Linear Mixed Models (GLMMs; *lme4* package; [38]) were used in all statistical analysis to account and control for pseudo-replication issues, because several birds were tracked during multiple years. Bird identity was included as a random term. GLMMs tested the effect of period (breeding *vs* non-breeding for each colony) and colony (Cima Islet *vs* Selvagem Grande for each period) on mean foraging trip characteristics, spatial ecology parameters and trophic signatures of Macaronesian shearwaters. The effect of sex was first included on the analysis, but removed after non-significant results were attained for this factor in all models (F < 1.23, P > 0.16). All variables were visually examined for normality (using Q-Q plots) and homoscedasticity (using Cleveland dotplots) before each statistical test, and transformed when necessary. All statistical analyses were performed using the software R. Results are given as means (±1 SD) with a significance level at p < 0.05.

## Results

### Spatial segregation

**Fig 1:**
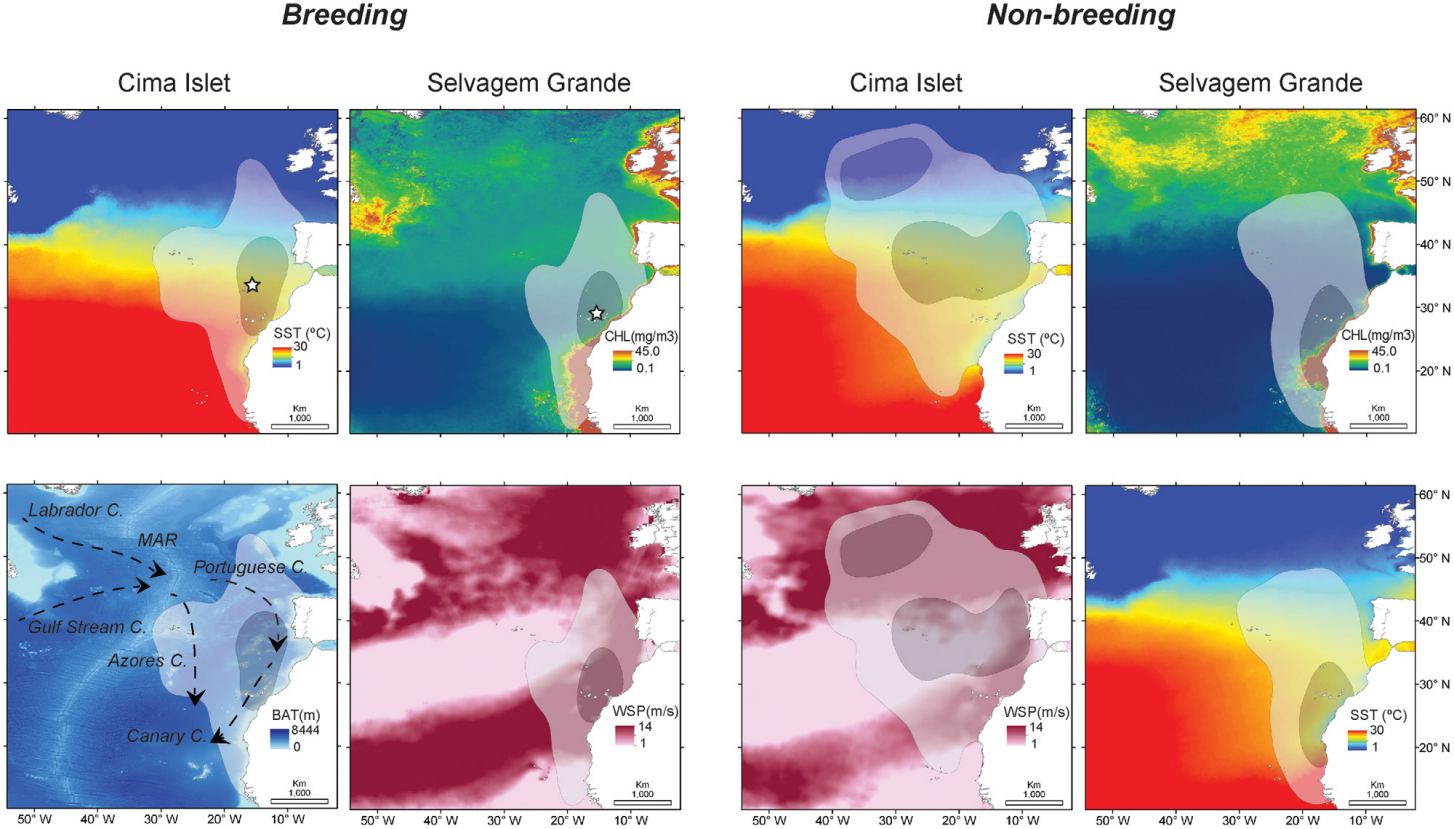
Breeding and non-breeding distribution of Macaronesian shearwater from Cima Islet and Selvagem Grande Island (white stars). Darker inner area—Kernel UD 50 and lighter outer area—Kernel UD 95 overlaid on Sea Surface Temperature (SST), sea surface chlorophyll *a* concentration (CHL), sea bathymetry (BAT) and wind speed (WSP). Dynamic predictors are shown as averaged composites for all the breeding (December-May) and non-breeding (June-November) periods. Also shown in the BAT map are the main Currents (e.g. Canary C.) influencing the oceanography of the study area. Selection of environmental predictors to show as background was (mostly) based on the importance of those variables for the four habitat niche (MaxEnt) models.

Macaronesian sharwaters did not undertake trans-equatorial migrations and maintained their foraging activity around the Madeiran archipelago, dispersing within the Azores, Portuguese and Canary Currents, until the Mid-Atlantic Ridge (MAR) region, on the north-west of Azores (Fig 1). All four habitat niche models showed a good to excellent ability to predict the observed habitat used by Macaronesian shearwaters (all AUC > 0.85). Overall, SST was the only environmental variable always important in explaining the habitat use by both populations (CI and SG), and during both periods (breeding and non-breeding). During the breeding phase, roughly from December to May (S1 Fig) birds from both populations (CI and SG) preferred an area closer to the breeding colony, between an oceanic area in the North of Madeira and the strong upwelling system at the African coast, thus mostly foraging within the Canary Current system. Spatial overlap during this phase, within and among populations was always higher than 76% (Table 1; S1 Fig). Both populations responded to cold and productive water regimes (SST, CHL), shallow (BAT) and sloppiest (BATG) areas and at close distance from their respective breeding colonies (DCOL; Table 2). During the non-breeding period, birds from SG kept targeting the productive Canary Current region, with a spatial overlap between phases always higher than 85% (S1 Fig). The same environmental variables kept their importance in explaining the habitats used by SG birds. Birds from CI dispersed and segregated from the SG population, mostly to an area extending until the Mid-Atlantic Ridge (MAR) and Labrador Current at the north-west of Azores and within the Azores and Portuguese Currents (Fig 1), with the spatial overlap among populations decreasing to less than 62% (Table 1; S1 Fig). Within this disparate and pelagic area, CI birds targeted different proxies of productivity, namely SST and CHL frontal regions (SSTG and CHLG), seamount areas (BAT) and windier regimes (WSPD; Table 2).

**Table 1:** Estimates of model fit and relative contributions of the environmental variables to the MaxEnt models generated for the spatial distribution of Macaronesian shearwaters from Cima Islet (Porto Santo) and Selvagem Grande. AUC—Area Under the Receiver Operating Curve.

	Breeding		Non-breeding	
	Cima Islet	Selvagem Grande	Cima Islet	Selvagem Grande
Test AUC (%)	91.3	92.8	85.5	89.4
**Parameter contribution (%)**				
Bathymetry (BAT; m)	**15.9**	**11.5**	—	**11.1**
Sea Surface Temperature (SST; °C)	**36.1**	**34.8**	**10.5**	**27.4**
Chlorophyll *a* concentration (CHL; mg m^-3^)	**23.1**	**25.7**	2.4	**19.2**
Gradient in BAT (BATG)	**12.1**	**11.8**	6.2	**13.8**
Gradient in SST (SSTG)	—	5.9	**17.3**	6.1
Gradient in CHL (CHLG)	—	—	**19.2**	—
Wind speed (WSPD; m s^-1^)	7.1	—	**40.3**	—
Distance to colony (DCOL; m)	**11.6**	**15.0**	6.6	**10.1**
**Permutation contribution (%)**				
Bathymetry (BAT; m)	45.5	7.6	—	25.7
Sea Surface Temperature (SST; °C)	23.4	6.8	5.9	18.6
Chlorophyll *a* concentration (CHL; mg m^-3^)	19.1	8.4	12.7	18.0
Gradient in BAT (BATG)	10.4	5.1	5.6	29.3
Gradient in SST (SSTG)	—	15.4	18.9	34.7
Gradient in CHL (CHLG)	—	—	20.8	—
Wind speed (WSPD; m s^-1^)	5.9	—	34.1	—
Distance to colony (DCOL; m)	27.6	36.3	15.7	11.5

**Table 2:** Mean foraging trip characteristics and trophic and spatial ecology parameters of Macaronesian shearwaters from Cima Islet (Porto Santo, N = 14 yearly trips) and Selvagem Grande (N = 9 yearly trips).

	Breeding		Non-breeding	
	Cima Islet	Selvagem Grande	Cima Islet	Selvagem Grande
**Foraging trip characteristics**				
Trip duration (d)	9.4 ± 3.7	4.8 ± 2.2	—	—
Proportion of daytime on the water surface (%)	19.5 ± 5.5	21.4 ± 7.2	53.0 ± 7.5	62.8 ± 8.0
Proportion of nighttime on the water surface (%)	11.7 ± 4.7	17.9 ± 5.8	59.2 ± 4.8	79.9 ± 5.7
**Spatial ecology parameters**				
HR overlaps within populations (%)	85.2 ± 8.3	91.3 ± 6.2	68.2 ± 7.7	95.3 ± 9.4
FR overlaps within populations (%)	86.4 ± 3.2	88.5 ± 9.3	53.2 ± 8.9	85.1 ± 11.1
HR overlaps among populations (%)	83.1 ± 4.2		52.1 ± 5.2	
FR overlaps among populations (%)	79.6 ± 5.2		7.8 ± 1.1	
**Habitat of foraging areas (50% Kernel UD)**				
Bathymetry (BAT; m)	556 ± 123	456 ± 166	1648 ± 230	409 ± 106
Sea Surface Temperature (SST; °C)	18.5 ± 0.9	19.9 ± 1.9	15.7 ± 0.5	18.3 ± 1.6
Chlorophyll *a* concentration (CHL; mg m^-3^)	1.09 ± 0.35	1.22 ± 0.56	0.21 ± 0.15	1.22 ± 0.56
Wind speed (WSPD; m s^-1^)	8.4 ± 1.6	9.0 ± 2.8	14.5 ± 1.9	10.3 ± 1.5
Distance to colony (DCOL; m)	665 ±248	389 ±105	2110 ± 598	410 ±105

### Behavioural segregation

During the breeding period, birds from CI made significantly longer foraging excursions (F_1,20_ = 8.12, P = 0.01, GLMM) than birds from SG. Birds from both populations spent similar amounts of daytime (F_1,20_ = 1.77, P = 0.21, GLMM) and nighttime (F_1,20_ = 1.65, P = 0.22, GLMM) on the water surface and had a similar home range (95% kernel UD: F_1,20_ = 2.75, P = 0.12, GLMM) and foraging regions (50% kernel UD: F_1,20_ = 2.88, P = 0.12, GLMM) overlap within populations. During the non-breeding phase, birds from CI travelled significantly more time, both during the day (F_1,20_ = 21.23, P < 0.001, GLMM) and at night (F_1,20_ = 17.99, P < 0.001, GLMM) than birds from SG. However, the percentage overlap within populations was significantly lower for CI birds than that of SG birds, either in terms of home range (HR: F_1,20_ = 19.71, P < 0.001, GLMM) and foraging regions (FR: F_1,20_ = 24.32, P < 0.001, GLMM; Table 1).

From the breeding to the non-breeding period, birds from CI significantly increased the proportion of daytime (F_1,12_ = 24.00, P < 0.001, GLMM) and nighttime (F_1,12_ = 32.66, P < 0.001, GLMM) on the water surface and, significantly decreased the HR (F_1,12_ = 41.14, P < 0.001, GLMM) and FR (F_1,12_ = 38.54, P < 0.001, GLMM) overlaps within populations. Birds from SG, significantly increased the proportion of daytime (F_1,6_ = 61.12, P < 0.001, GLMM) and nighttime (F_1,6_ = 58.90, P < 0.001, GLMM) on the water surface, while exhibiting similar HR (F_1,6_ = 2.01, P = 0.21, GLMM) and FR (F_1,6_ = 2.34, P = 0.17, GLMM) overlaps between periods. Between the breeding and the non-breeding phases there was a significant decrease in both the HR (F_1,20_ = 17.09, P < 0.01, GLMM) and FR (F_1,20_ = 22.77, P < 0.001, GLMM) overlaps between populations (Table 1).

**Fig 2:**
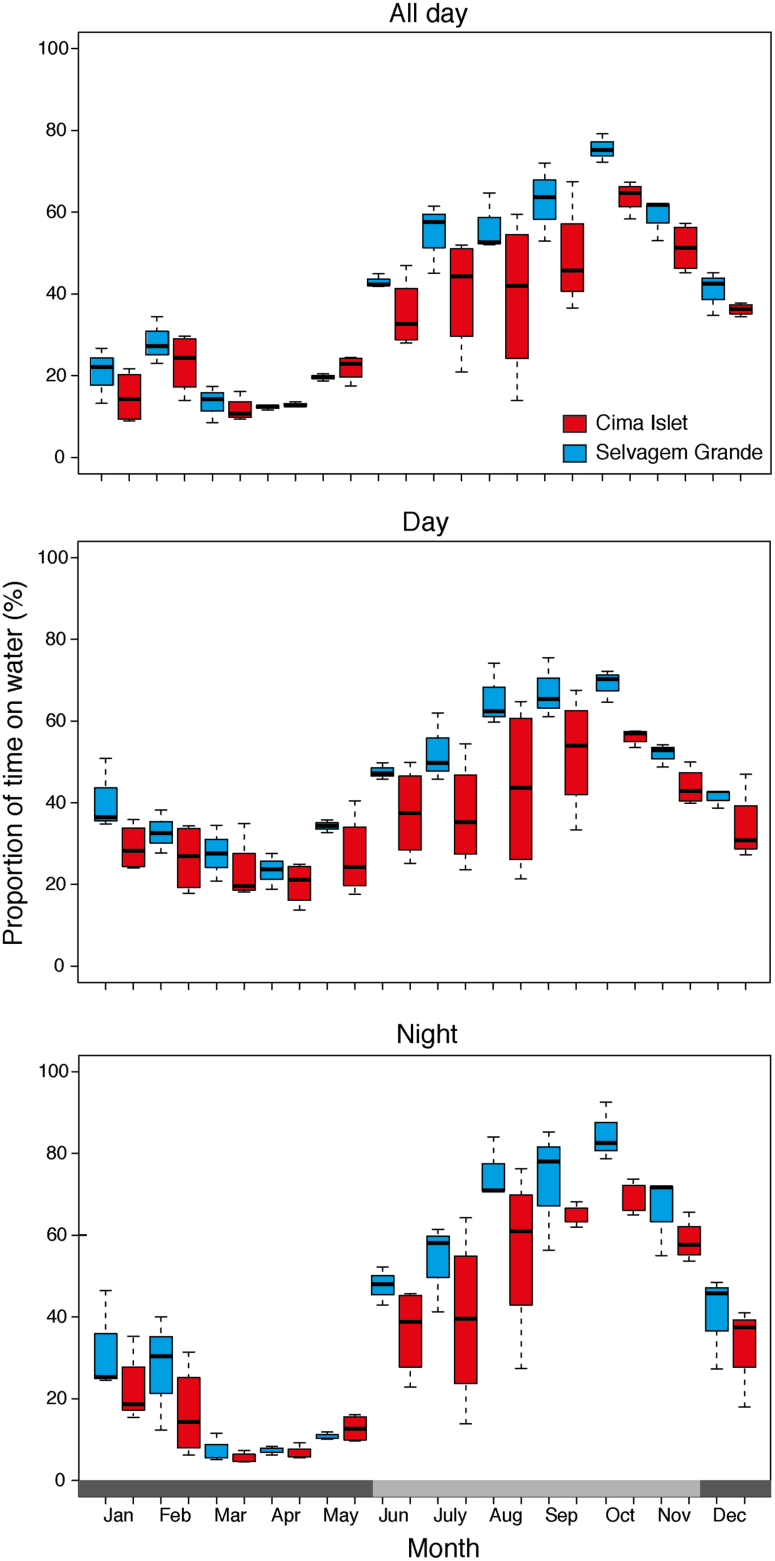
Median, 25–75% inter-quartile range, non-outlier range, and outliers of the proportion of time in water for birds from Cima Islet (dark grey) and Selvagem Grande (light grey).

The proportion of time spent on the water (i.e. a proxy of at-sea activity) varied greatly during the year and day period for both populations, though the activity of CI birds varied more (maximum monthly range = 43% for July during the night) than that of SG birds (maximum monthly range = 29% for September also during the night). Mostly during the non-breeding period, it was evident the segregation on the at sea activity between populations in all three day periods considered (i.e. all day, daytime and nighttime) (Fig 2).

### Trophic segregation

**Fig 3:**
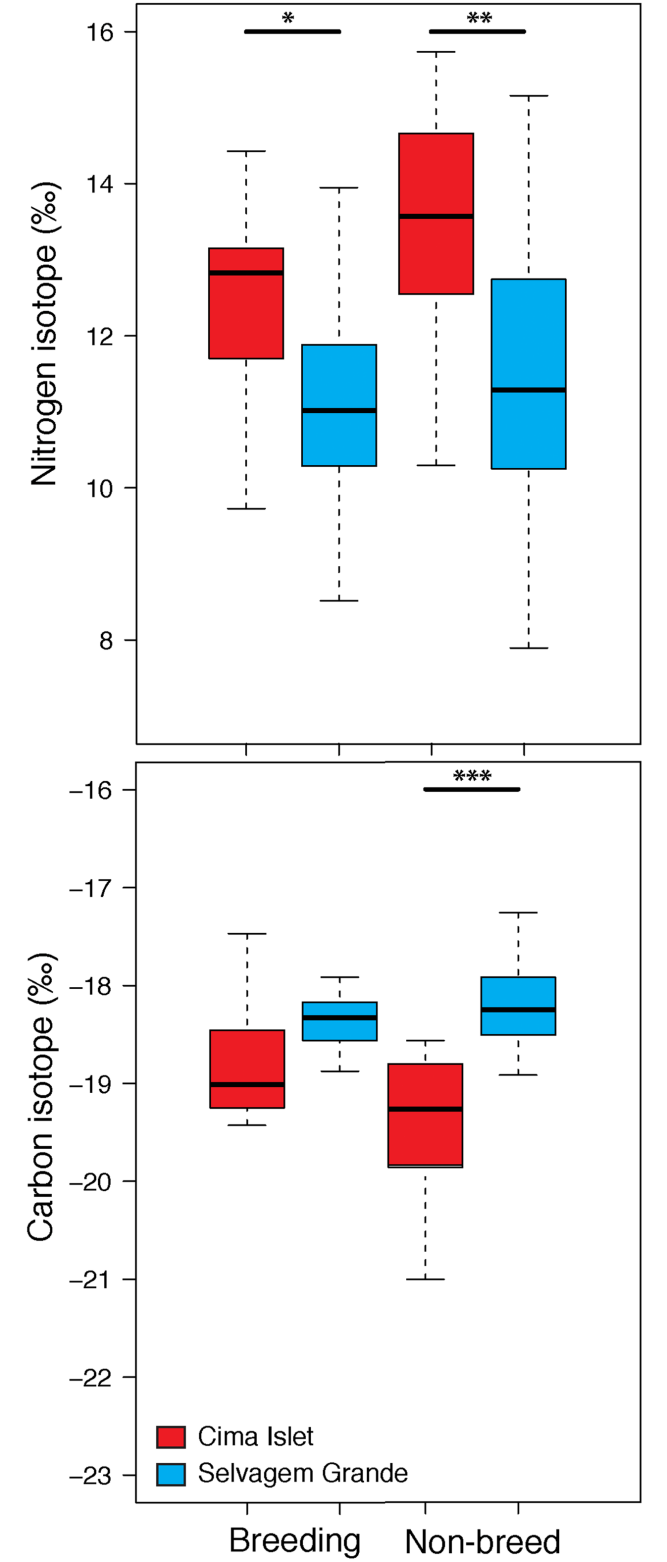
(A) Nitrogen and (B) carbon isotopic signatures (median, 25–75% inter-quartile range, non-outlier range, and outliers) of birds from Cima Islet (CI; red) and Selvagem Grande (SG; blue) Islands. Sample sizes corresponded to the amount of devices retrieved each year, with 4, 2 and 2 feather samples from SG and 3, 4 and 3 from CI, respectively in 2011, 2012 and 2013. Breeding—innermost primary feathers and, Non-breeding—eighth secondary feathers. Statistical significance: * p < 0.05, ** p < 0.01, *** p < 0.001.

Birds from CI had systematically an enriched nitrogen signature than their SG congeners, during breeding (P1 feathers: F_1,20_ = 6.41, P = 0.02, GLMM) and non-breeding (S8 feathers: F_1,20_ = 7.08, P < 0.01, GLMM) periods. The carbon signature of CI birds was significantly lower than that of SG birds during the non-breeding (S8 feathers: F_1,20_ = 19.82, P < 0.001, GLMM) period (Fig 3).

## Discussion

Our study suggests that similarly to other procellariiform species, the distribution of these two Macaronesian shearwater sub-populations does not represent a random dispersal (both during the breeding and non-breeding periods), but in fact suggests a strong degree of segregation in some ‘sub-population-specific’ regions [35]. One hypothesis for such pattern is that each sub-population have evolved and adapted to feed on particular and ‘sub-population-specific’ resources, and the segregation observed at the three different levels (spatial, behavioural and trophic) might in fact be a result of such adaptation. This may apply for long-lived and philopatric populations in which the use of the same foraging regions in repeated trips and year after year, might lead to the emergence of ‘cultural foraging patterns’ [39]. Below we discuss the intriguing at-sea behaviour of both sub-populations, breeding just ~340 km apart, but segregating in the use of space during the non-breeding phase, while closely resembling on their foraging and behavioural patterns during the breeding stage.

### Spatial segregation

Both populations exhibited similar foraging patterns and high spatial overlap during the breeding phase (winter—spring), related with their ‘central-place foraging behaviour’, having to commute between patches of high prey availability and their colony, either to incubate the egg or to provision their chick [3]. This should explain the importance reached by the DCOL variable on the habitat niche models. In practice, because the immediate surroundings of both colonies, CI and SG, are relatively low productive [28], birds exploited the closer most productive region, the Canary Current ecosystem. On the other hand, breeding in winter-spring, instead of spring-summer, as the majority of the larger procellariiform species in the north Atlantic area might be a strategy to avoid inter-specific competition with Bulwer’s petrels *Bulweria bulwerii* and Cory’s shearwaters for breeding burrows [40] and at-sea food resources with larger and similar sized seabird species [14]. Nonetheless, we can’t exclude the hypothesis that Macaronesian shearwaters might avoid at-sea competition for resources by preying on species less targeted by other seabirds, or preys belonging to differentiate trophic chains and niches [41]. For instance, Cory’s shearwater populations also breed (broadly between March and November) on Cima Islet and Selvagem Grande and intensively forage within the Canary Current system [3,7,42]. However, the same Canary Current region (i.e. north-west coast of Africa) is also heavily used as a foraging ground by thousands of wintering and migrating seabirds during autumn-winter. For instance, northern gannets *Morus bassanus* [43,44], Scopoli’s shearwaters *Calonectris diomedea* [45,46], and large numbers of different storm-petrel, skuas and phalarope species [47], take advantage of the intense upwelling and fishery activity (i.e. feeding on discards) existent on the area.

When relieved from breeding duties, seabirds no longer behave as central-place foragers, thus dispersing/ migrating usually following the seasonal productivity patterns in neritic and open ocean systems (e.g. [48]). Interestingly, during this phase birds from CI dispersed much more than birds from SG, thus investing on a completely different foraging strategy and spatially segregating from their SG conspecifics. One hypothesis for this disparate pattern might be related with avoiding competition with other larger seabird species at the Canary Current region. For instance, during spring—autumn this area holds large numbers of foraging Cory’s shearwaters, breeding in Madeira, Selvagens, Canary [3,7] and Berlengas archipelagos [49,50]. While birds from SG remained exploiting the productive waters within the Canary Current system, birds from CI foraged mostly between the Azores and Portuguese Currents and on the Mid-Atlantic Ridge (MAR) area with a very small overlap in distribution between Madeiran populations. Noticeably, at the MAR region CI birds overlapped in their distribution with their conspecifics from Azores, mostly exploiting deep water grounds within that region [12]. The MAR area also holds other seabird species when both populations of Macaronesian shearwaters are still breeding, and thus foraging mostly within the Canary Current region. Thus, between October—May the area holds, for instance, large numbers of Kittiwakes *Rissa tridactyla* [51,52], Cory’s shearwaters [53,54], Zino’s *Pterodroma madeira* [55] and Desertas *Petrodroma deserta* [56] petrels, thus partially coinciding spatially and temporally with Macaronesian shearwaters from CI.

Sea Surface Temperature (SST) was one of the most important environmental predictors of habitat suitability in all four habitat niche models built for Macaronesian shearwaters. This is in line to what happens in the majority of research studies linking at-sea seabird positional information with marine environment characteristics (see review by [57]). During the breeding phase, both CI and SG populations exploited the Canary Current system and neritic area of the African shelf where shallow foraging grounds (low BAT), productive regimes (higher CHL, lower SST) and steeper areas (BATG; usually interpreted as areas of local upwelling phenomena) dictate higher productivity patterns [58]. Cory’s shearwaters [42] and Desertas petrels [56] breeding on Selvagens and Madeira archipelagos (respectively) seem to respond to similar environmental cues when foraging over the same region.

**Fig 4:**
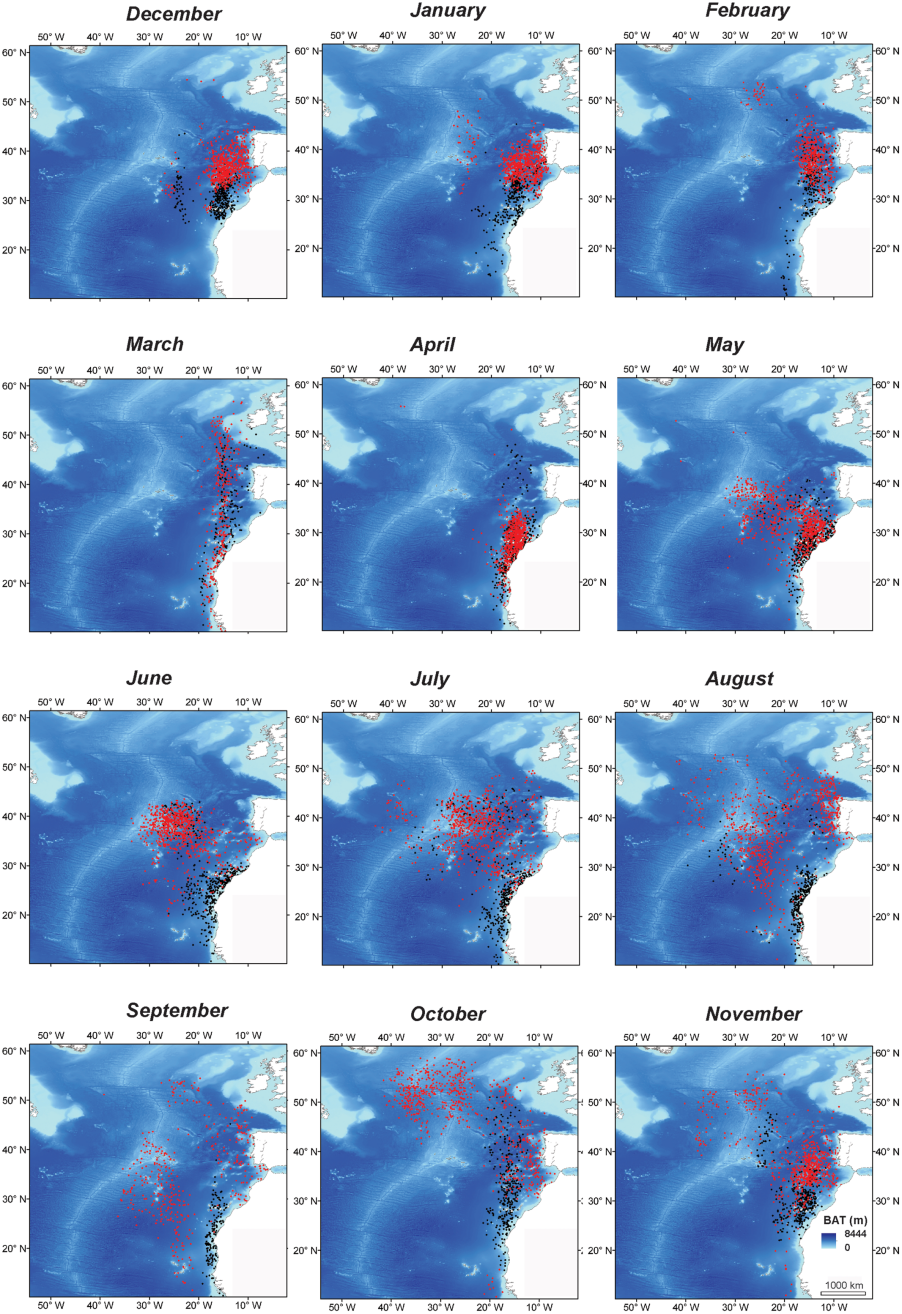
Monthly distribution of tracked Macaronesian shearwaters breeding on Cima Islet (Porto Santo; red dots; N = 14 trips) and Selvagem Grande (black dots; N = 9 trips), during their breeding (December—May) and non-breeding (June—November) phases.

During the non-breeding period and within the Azores and Portuguese Currents and MAR area, birds from CI concentrated their foraging effort on regions with known seamounts and upwelling phenomena, which are natural enhancers of productivity and congregators of preys [59]. Such areas are prolific in local eddies, fronts and upwelling phenomena promoted by known seamounts and banks of this region [5]. This was evident on the habitat niche model of birds from CI during non-breeding, when environmental triggers of habitat use were mostly related with gradients of SST and CHL, which are seen as proxies of eddies and frontal regions (e.g. [42]). Similarly, Desertas petrels *Pterodroma deserta* exploit this region mostly during their breeding period (summer—autumn), coinciding spatial and temporally with birds from CI and also responding to proxies of pelagic frontal regimes (both SSTG and CHLG; [56]). Furthermore, while exploiting pelagic areas in central north-Atlantic, birds from CI took advantage of oceanic wind fields (i.e. WSPD on the habitat niche models), to propel with minimum energetic costs their foraging excursions in the open ocean [60]. Moreover, the monthly movement of the CI birds during the non-breeding season, firstly towards the MAR area, in the open ocean, and then descending via the Portuguese coast, strongly suggests that birds were using prevailing winds on this long-distance movements (Fig 4).

### Behavioural and trophic segregation

For both populations the foraging effort (i.e. time spent flying) was greater during the active breeding than during the non-breeding phase. Activity patterns clearly reveal the extra effort (i.e. more time spent flying, at higher speed and through longer distances) that birds have to do during the breeding period to incubate the egg and rear their chick. These results are in line with those obtained for Macaronesian shearwaters populations from Azores [12] and related seabird species inhabiting the same area, such as the Desertas petrel [56]. In both studies, breeders noticeably increased their flying time in response to their chick needs at the colony and/ or fulfilling their own energetic requirements while incubating the egg or rearing the chick. When relieved from their breeding duties (i.e. during the non-breeding phase), birds only need to find food resources for themselves and thus can spend longer periods resting on the water surface. Noticeably, Macaronesian shearwaters from Azores spend an even higher proportion of time in the water surface both during daytime and nighttime [12], when compared to both CI and SG birds, thus showing some degree of behavioural plasticity among populations.

Nonetheless, some behavioural differences among sub-populations were obvious from the results. Markedly, birds from CI had higher proxies of foraging effort than birds from SG, mostly during the non-breeding phase, but even while breeding (and thus exploiting similar habitats). This should be related with the fact that both sub-populations were foraging on a similar region (the CC system), but birds from CI have to travel longer distances to reach this region departing from their colony, when compared to the more proximate location of SG birds in relation to the coast of Africa. During the non-breeding period, CI birds flew more time in search for food over the Azores and Portuguese Currents when compared to SG birds foraging over the CC system. Such higher foraging effort of birds from CI seems to be compensated by preying on nitrogen enriched prey, as shown by their persistently higher nitrogen signature when compared to SG individuals.

In the ocean, the distribution of nitrogen isotopes varies geographically [61], which directly shapes the trophic niche of prey inhabiting a specific location [62] and predators feeding on those prey [63]. These baseline isotopic landscapes (i.e. isoscapes), associated with the existent spatial segregation, might have been the drivers of the trophic segregation among CI and SG individuals (specially on the nitrogen isotopic values). Nevertheless, both sub-populations were isotopically segregated even during phases when they were foraging in practically the same regions (i.e. during the breeding period), which may be due to individuals feeding on isotopically different prey [64]. Carbon isotopic values usually segregates consumers feeding habits in coastal environments (more enriched) from pelagic habitats (more depleted; [63]). The exploitation of pelagic areas by CI birds, might have shaped the lower carbon signature of their secondary eight feather moulted during the non-breeding phase, and thus segregated isotopically from their SG conspecifics [13]. However, SG birds exploiting a coastal environment all year round, exhibited carbon isotopic values more proximate to the carbon levels of Macaronesian shearwaters from Azores, which only exploit pelagic environments [12,65]. Like discussed by [12], the relatively high inter-annual variability on the isotopic values (both carbon and nitrogen signatures) of the Macaronesian population from Azores, may be due to (1) shifts on the abundance and availability of a given prey with different isotopic values (when compared to the usual dietary choices of the species) or (2) differences on the baseline values of the isoscape (carbon and nitrogen isotopes) driven by oceanographic or climate changes. Thus, the apparent trophic segregation between CI and SG sub-populations should be interpreted with cautious, because the species has a relatively high dietary plasticity [12], the isoscapes at the north-east Atlantic region are generally homogeneous [66] but prone to changes due to environmental stochasticity and the isotopic values had a large (generally overlapping) range (our study), which may be indicative of a high inter-annual variability on the dietary choices, rather than ‘pure’ trophic segregation between sub-populations. In fact, diet samples from individuals of the same colonies showed inter-annual variation in the diet composition; with birds from CI showing higher proportion of cephalopods in their diet in 2011 and lower proportion of fish prey in 2012, when compared to SG individuals [13].

### Conservation implications

Our results highlight the importance of the Canary Current (CC) ecosystem for this species, in particular the Western Sahara and Morocco continental shelves. Since these areas undergo high fishing pressure, there is a need to understand the direct (i.e. by-catch) and indirect (i.e. competition for food) impacts of fishing activities on this Macaronesian endemic species. This may be important to understand the strong decline in the number of fledglings in Gran Canaria (Canary Islands) since the 90’s (~75% decrease) [67]. Monitoring secondary production levels within the hotspots delineated in this work (core foraging regions for both CI and SG populations) may be a potentially useful step to better understand population declines. Only a long term monitoring scheme (both tracking and studying the trophic choices of individuals) will clarify if these patterns of sub-population-level segregation are maintained, when facing years of contrasting environmental conditions and a predicted global warming phenomenon.

## Supporting Information

S1 Fig Distribution of individual Macaronesian shearwater. Breeding and non-breeding distribution (50% kernel Utilization Distribution) of individual Macaronesian shearwater *Puffinus baroli*. Colours represent different individuals.
(DOCX)
