# Supplementary material for: Population-Scale Foraging Segregation in an Apex Predator of the North Atlantic
Source: PLoS One. 2016 Mar 22;11(3):e0151340. doi: 10.1371/journal.pone.0151340 (PMC4803222; doi:10.1371/journal.pone.0151340)

**S1 Fig.** Breeding and non-breeding distribution (50% kernel Utilization Distribution) of individual Macaronesian shearwater *Puffinus baroli*. Colours represent different individuals.


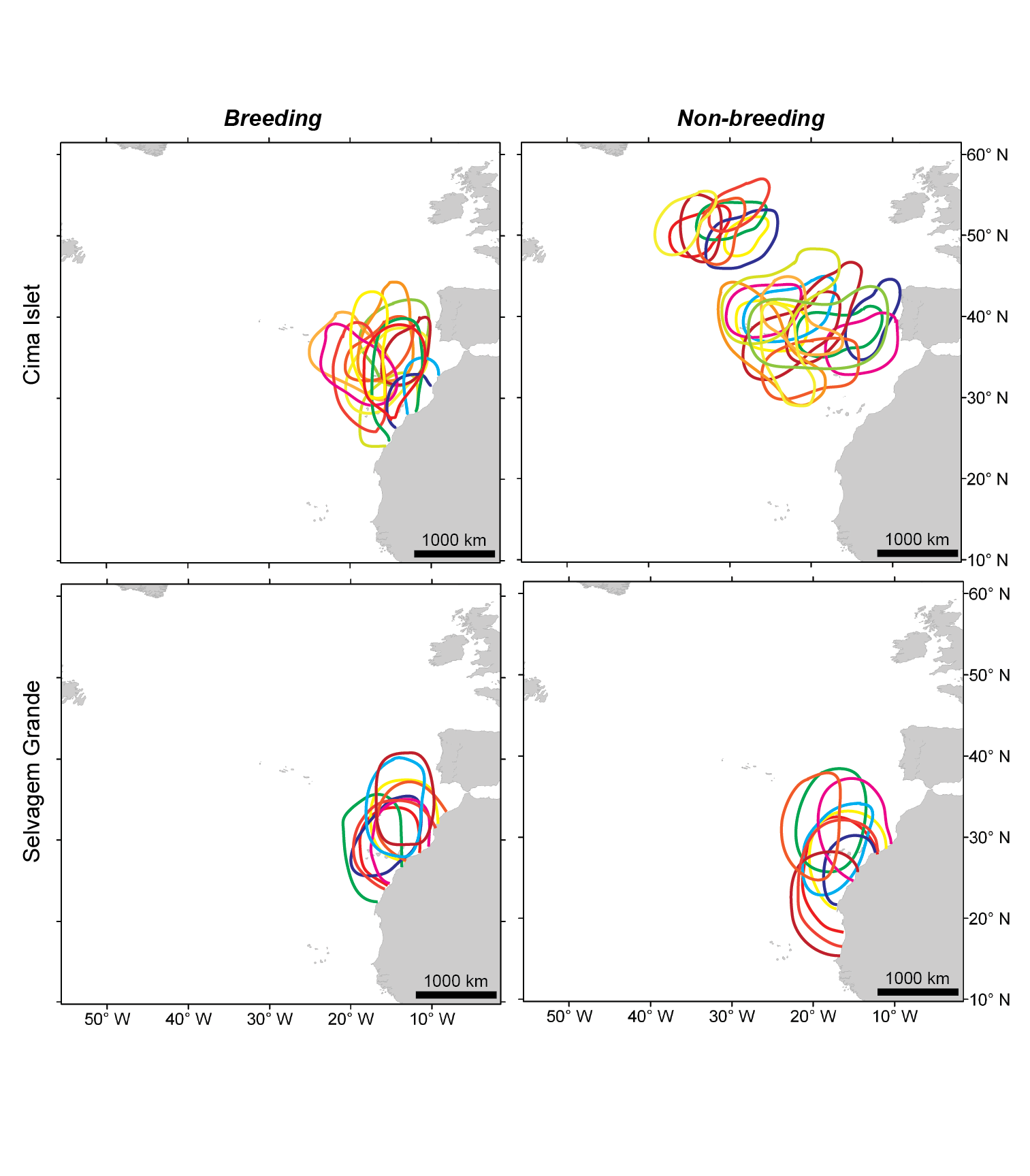

Supplement: S1 Fig — Breeding and non-breeding distribution (50% kernel Utilization Distribution) of individual Macaronesian shearwater Puffinus baroli. Colours represent different individuals. (DOCX) [file pone.0151340.s001.docx]
